# Item

**Published:** 1898-12

**Authors:** 


### ITEM.

Dr. J. L. C. Cronyn, of Buffalo, has designed a sanitary candle, intended for army, hospital and private uses. This candle will afford light as well as disinfection, is made of paraffin, mixed with antiseptic ingredients, and can be supplied at a nominal price, thus bringing it within the reach of all.

